# Augmentation of the therapeutic efficacy of WEE1 kinase inhibitor AZD1775 by inhibiting the YAP–E2F1–DNA damage response pathway axis

**DOI:** 10.1002/2211-5463.12440

**Published:** 2018-05-22

**Authors:** Yusuke Oku, Naoyuki Nishiya, Takaaki Tazawa, Takaya Kobayashi, Nanami Umezawa, Yasuyo Sugawara, Yoshimasa Uehara

**Affiliations:** ^1^ Department of Integrated Information for Pharmaceutical Sciences Iwate Medical University School of Pharmacy Yahaba‐cho Japan

**Keywords:** AZD1775, combination therapy, dasatinib, DNA damage, YAP

## Abstract

The main reasons for failure of cancer chemotherapy are intrinsic and acquired drug resistance. The Hippo pathway effector Yes‐associated protein (YAP) is associated with resistance to both cytotoxic and molecular targeted drugs. Several lines of evidence indicate that YAP activates transcriptional programmes to promote cell cycle progression and DNA damage responses. Therefore, we hypothesised that YAP is involved in the sensitivity of cancer cells to small‐molecule agents targeting cell cycle‐related proteins. Here, we report that the inactivation of YAP sensitises the OVCAR‐8 ovarian cancer cell line to AZD1775, a small‐molecule WEE1 kinase inhibitor. The accumulation of DNA damage and mitotic failures induced by AZD1775‐based therapy were further enhanced by YAP depletion. YAP depletion reduced the expression of the Fanconi anaemia (FA) pathway components required for DNA repair and their transcriptional regulator E2F1. These results suggest that YAP activates the DNA damage response pathway, exemplified by the FA pathway and E2F1. Furthermore, we aimed to apply this finding to combination chemotherapy against ovarian cancers. The regimen containing dasatinib, which inhibits the nuclear localisation of YAP, improved the response to AZD1775‐based therapy in the OVCAR‐8 ovarian cancer cell line. We propose that dasatinib acts as a chemosensitiser for a subset of molecular targeted drugs, including AZD1775, by targeting YAP.

**Abbreviations**
CDK: cyclin‐dependent kinase
EGFR: epidermal growth factor receptor
FA: Fanconi anaemia
FDA: Food and Drug Administration
PARP: poly(ADP‐ribose) polymerase
TAZ: transcriptional activator with PDZ‐binding motif
YAP: Yes‐associated protein

Although molecular targeted therapies are widely beneficial to patients with cancer, a significant number of patients do not obtain benefits. Acquired drug resistance of tumour cells to molecular targeted drugs, especially kinase inhibitors, is a major concern in cancer therapy. Therefore, to maximise the efficacy of molecular targeted drugs and prevent tumour cells from building resistance to monotherapy, rational drug combinations have been proposed on the basis of augmentation effects between drugs 1.

The Hippo pathway transducers, Yes‐associated protein (YAP) and transcriptional coactivator with PDZ‐binding motif (TAZ), are involved in various cellular processes, including organ size control and tissue regeneration. Aberrant expression of YAP or TAZ induces solid tumours in multiple tissues 2 and imparts resistance to cytotoxic anticancer drugs to tumour cells in various cancers 3, 4, 5. Recent studies have demonstrated that YAP and TAZ are also involved in resistance of tumour cells to molecular targeted drugs 6, 7, 8, 9. As there are crosstalks between the Hippo and other pathways like the mitogen‐activating kinase pathway 10, YAP might be involved in the sensitivity of tumour cells to molecular targeted drugs, including those under preclinical or clinical evaluations.

Yes‐associated protein promotes cell cycle progression by controlling gene expressions. For instance, YAP activation reverses cell cycle arrest of mature hepatocytes lacking Rb family genes through the expression of cell cycle genes 11. YAP–TEAD4 interaction activates cyclin‐dependent kinase (CDK)–cyclin expression and inactivates CDK inhibitors 12. YAP–TEAD2 binds to E2F1 and promotes cell cycle progression and DNA replication 13. In mesothelioma cell lines, YAP is required for the expression of *E2F1* and other cell cycle‐related genes 14. Therefore, we hypothesised that YAP is associated with sensitivity of tumour cells to drugs targeting cell cycle‐related proteins.

Upon DNA damage in the S and G2 phases, WEE1 kinase induces cell cycle arrest through the inhibitory phosphorylation of CDK1 and CDK2 15, preventing premature entry of cells into mitosis. The inhibition of WEE1 promotes premature entry of cells into mitosis from S or G2 phases 16, 17. AZD1775 (formally MK1775) is a potent and selective small‐molecule kinase inhibitor of WEE1 18. In various models of cancer, when combined with different classes of DNA‐damaging drugs, such as gemcitabine, platinum drugs, topoisomerase inhibitors and the poly(ADP‐ribose) polymerase (PAPR) inhibitor, AZD1775 increases DNA damage and induces catastrophic mitosis 19, 20, 21, 22, 23.

Several determinants of AZD1775 sensitivity have been identified. AZD1775 selectively kills H3K36me3‐deficient cancers 24. In addition, depletion of the DNA damage response genes including the components of Fanconi anaemia (FA) or a homologous recombination DNA repair pathway leads to increased sensitivity of tumour cells to AZD1775‐based therapy in colon and breast cancers 25.

In the current study, we showed that YAP inactivation sensitises the OVCAR‐8 ovarian cancer cell line to the AZD1775‐based therapy. YAP inactivation further increases DNA damage and mitotic failures induced by AZD1775–gemcitabine combination therapy. In addition, the YAP depletion leads to the decrease in expression of DNA damage response proteins exemplified by FA components, and their transcriptional regulator E2F1. As an application of our findings, we combined AZD1775–gemcitabine with dasatinib which inhibits the nuclear localisation of YAP 5, 26. Similar to the effects of YAP depletion, this combination therapy efficiently increased cell death and induces DNA damage. These results give insights into a potential combination therapy for ovarian cancers that impairs the DNA damage response through the YAP–E2F1–DNA damage response pathway axis. Dasatinib might act as chemosensitiser to subsets of molecular targeted drugs by inhibiting YAP/TAZ.

## Materials and methods

### Cell culture and treatments

OVCAR‐8 cells were maintained in RPMI‐1640 medium containing 10% FBS and penicillin/streptomycin. Dasatinib was purchased from JS Research Chemicals Trading Co. (Wedel, Germany), gemcitabine from Wako Pure Chemicals (Osaka, Japan) and AZD1775 and olaparib from Adooq Bioscience (Irvine, CA, USA). For a single thymidine block, the cells were treated with 2 mm of thymidine for 24 h and released into a fresh medium with or without drugs for 12 h.

### RNAi

siRNA transfection was performed using Lipofectamine RNAiMAX (Thermo Fisher Scientific, Waltham, MA, USA) according to the manufacturer's instructions. A GFP‐specific siRNA was purchased from Nippon Gene (Toyama, Japan) and used as a negative control. The siRNA species for YAP were as follows: #1: GACAUCUUCUGGUCAGAGAUU 3, #2: GGUGAUACUAUCAACCAAATT (Thermo Fisher Scientific) and #3: AGAGAUACUUCUUAAAUCATT (Thermo Fisher Scientific).

### Western blotting

Western blotting was performed by standard methods. The following antibodies were used: 1/3000 rabbit anti‐YAP/TAZ (Cell Signaling Technology, Danvers, MA, USA; #8418), 1/10 000 mouse anti‐GAPDH (EMD Millipore, Burlington, MA, USA; MAB374), 1/3000 rabbit PAPR (Cell Signaling Technology; #9542), 1/5000 mouse anti‐E2F1 (Proteintech, Rosemont, IL, USA; 66515‐1‐Ig), 1/2000 rabbit anti‐FANCD2 (Proteintech; 204006‐1‐AP), 1/3000 mouse anti‐epidermal growth factor receptor (EGFR; Cell Signaling, #2239), 1/5000 anti‐mouse IgG‐HRP (GE Healthcare, Buckinghamshire, UK) and 1/5000 anti‐rabbit IgG‐HRP (GE Healthcare). All antibodies were diluted with Can Get Signal reagent (TOYOBO, Osaka, Japan). The band intensity was quantified by imagej (National Institute of Health, Bethesda, MD, USA), and the percentage of cleaved PARP was calculated as the percentages of cleaved PARP in total PARP: cleaved PARP/(full‐length PARP + cleaved PARP) × 100 in each sample.

### MTT assay

Around 7000 cells suspended in RPMI‐1640 medium containing 10% FBS were seeded on a 96‐well plate. The cells were incubated in the presence of drugs for 4 days. Then, 0.5 mg·mL^−1^ of 3‐(4,5‐dimethylthiazol‐2‐yl)‐2,5‐diphenyltetrazolium bromide (MTT; Sigma‐Aldrich, St. Louis, WA, USA) was added, and the solution was incubated again for 4 h. Formazan was solubilised by 8% sodium dodecyl sulfate overnight. The optical density at 570 nm was measured by a microplate reader (Molecular Devices, San Jose, CA, USA).

### Colony formation assay

About 50 000 cells were seeded on 24‐well plates and indicated concentration of AZD1775 with or without 1.5 nm gemcitabine for 4 days. Cells were fixed with 4% paraformaldehyde and stained with 0.5% crystal violet.

### Flow cytometry

The cells were washed with PBS and stained with annexin V‐allophycocyanin (annexin V‐APC; BioLegend, San Diego, CA, USA) and 10 μg·mL^−1^ of propidium iodide for 15 min. A total of 30 000 cells were analysed by FACSAria (GE Healthcare) using facsdiva software.

### Immunofluorescence and imaging

The cells were fixed with ice‐cold methanol for 15 min for staining γ‐H2AX and with 4% paraformaldehyde for staining mitotic cells and YAP/TAZ, respectively. The fixed cells were permeabilised with 0.3% Triton X‐100 in PBS and blocked with 3% fetal bovine serum in PBS for 30 min. They were incubated with 1/1000 rabbit anti‐γ‐H2AX (Genetex, Irvine, CA, USA; GTX127340), 1/2000 mouse anti‐α‐tubulin (Sigma‐Aldrich; B‐5‐1‐2), 1/500 anti‐phospho‐Histone H3 S10 (Cell Signaling Technology; #9701) or 1/300 rabbit anti‐YAP/TAZ antibody (Cell Signaling Technology; #8418) at 4 °C overnight and then washed with PBS three times. Next, the cells were incubated with 1/500 anti‐rabbit IgG‐Alexa Fluor 488, 1/500 anti‐mouse IgG‐Alexa Fluor 488, anti‐rabbit IgG‐Alexa Fluor 594 or phalloidin‐Alexa Fluor 594 (Thermo Fisher) for 1 h at room temperature and again washed with PBS three times. Then, the cells were mounted in Prolong Diamond reagent containing 10 μg·mL^−1^ of Hoechst 33342 (Thermo Fisher Scientific). Images were obtained with an FV1000‐D confocal microscope using fv10‐asw software (Olympus, Tokyo, Japan). To capture mitotic cells, ~ 40 images were collected with a *z*‐optical spacing of 0.2 μm with a 100× numerical aperture 1.4 objective lens. For other cells, single sections were collected with a 40x objective lens.

### Quantitative real‐time PCR

Total RNA was isolated with ISOGEN (Nippon Gene), and cDNA was synthesised with the ReverTra Ace qPCR RT master mix with gDNA remover (TOYOBO). RT‐PCR was performed with the THUNDERBIRD SYBR qPCR Mix (TOYOBO) using the Eco Real Time PCR system (Illumina, San Diego, CA, USA). The sequences of the PCR primers for *FANCD2*,* FANCA*,* FANCG* and *E2F1* were as follows: FANCD2‐F: CAAACAGAATGAAGCCAGCA; FANC D2‐R: CCATGGTCACAGCACCAATA; FANCA‐F: GTTGCCTCTAGCGTGGGAC; FANCA‐R: GGAGAACATACTGTGTGCCAAT; FANCG‐F: TAGGCTCTATCAGCAACTGGG; FANCG‐R: AAACTGCGGGGCTTT GGAA; E2F1‐F: CATCCCAGGAGGTCACTTCTG; and E2F1‐R: GACAACAGCGGTTCTTGCTC; and the *RPL13A* primer was purchased from Qiagen (Hilden, Germany).

### Statistical analysis

The MTT assay, γ‐H2AX‐positive cells, chromosome segregation defect and qPCR data are shown as mean and standard deviation. Comparison between two groups was performed using an independent‐sample *t‐*test. A *P*‐value of < 0.05 was considered statistically significant.

## Results

### YAP inactivation sensitises OVCAR‐8 ovarian cancer cells to AZD1775‐based therapy

**Figure 1:**
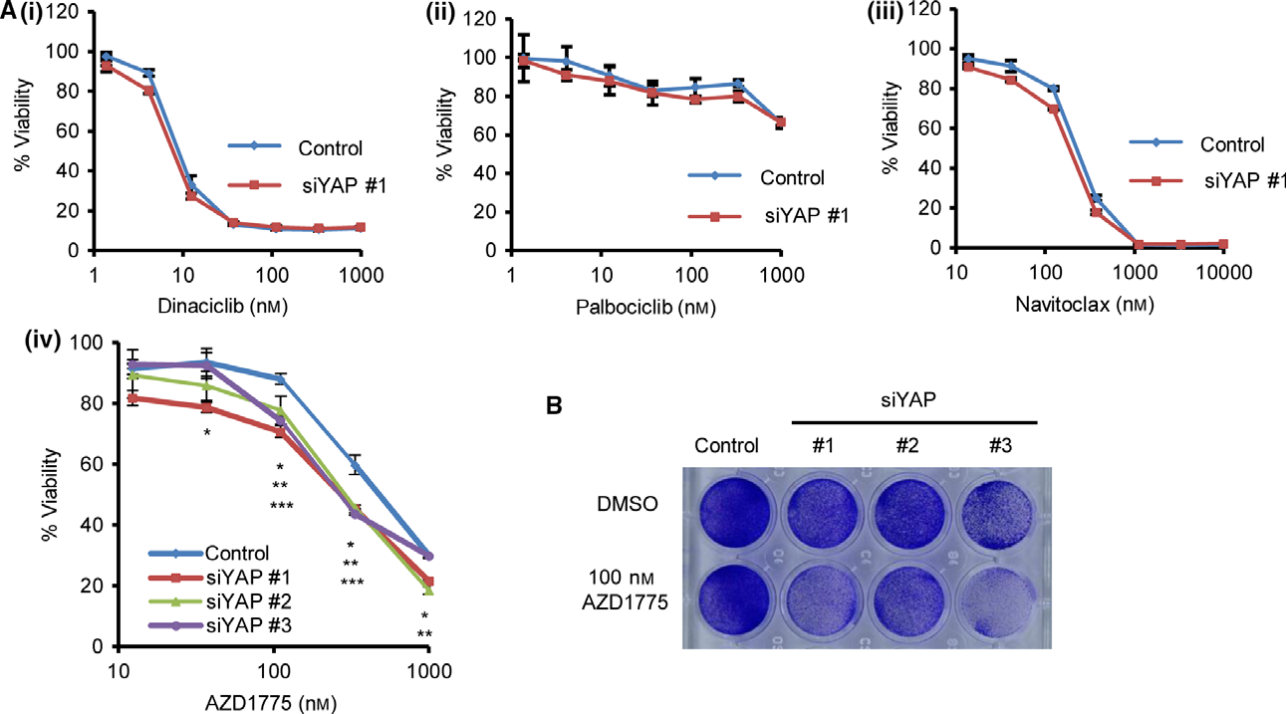
YAP inactivation sensitises OVCAR‐8 ovarian cancer cells to AZD1775‐based therapy. (A) YAP was depleted using siRNA, and the cells were treated with drugs for 4 days. Viability was measured using an MTT assay. (i) dinaciclib (a CDK inhibitor), (ii) palbociclib (a CDK4/6 inhibitor), (iii) navitoclax (a BCL family inhibitor), (iv) AZD1775. The viability was measured using the absorbance without AZD1775 as 100% in each sample. The data are representative of at least two independent experiments. Significant differences between the groups were evaluated using an independent‐sample *t*‐test. *P <* *0.05 (control versus siYAP #1), **P <* *0.05 (control versus siYAP #2), ***P <* *0.05 (control versus siYAP #3). (B) YAP was depleted using three different siRNA species, and cells were treated with 100 nm AZD1775 for 4 days. Cells were fixed and stained with 0.5% crystal violet.

**Figure 2:**
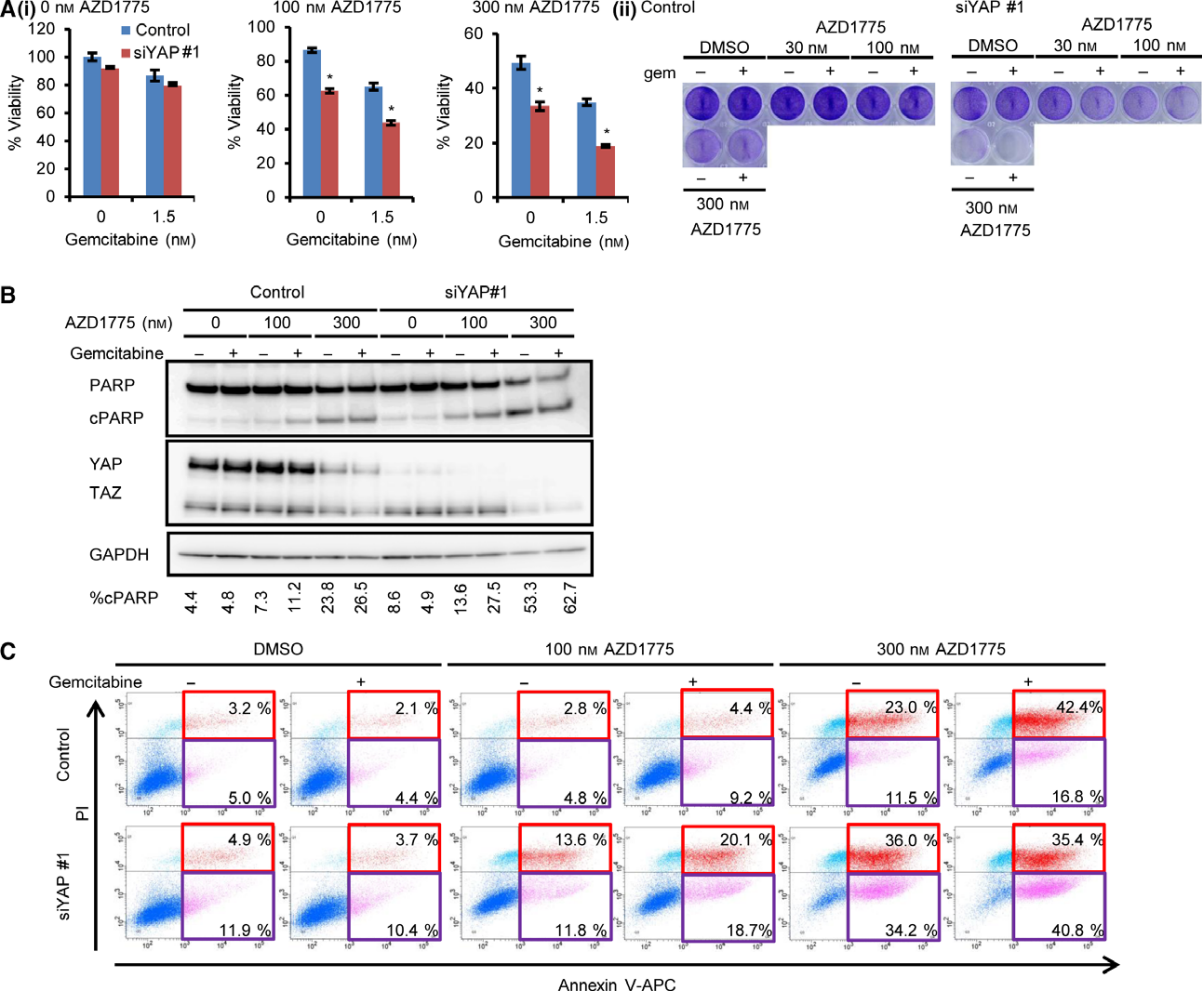
Inactivation of YAP sensitises the OVCAR‐8 ovarian cancer cells to AZD1775–gemcitabine combination therapy. (A) (i) YAP was depleted using siRNA, and the cells were treated with the indicated concentrations of AZD1775 and gemcitabine for 4 days. Viability was measured using MTT assay. The viability was measured using the absorbance without AZD1775 as 100% in each sample. The data are representative of at least three independent experiments. Significant differences between the groups were evaluated using an independent‐sample *t*‐test. *P <* *0.05. (ii) Colony formation assay. Cells were treated with siRNA, and next day, cells were treated with indicated concentration of AZD1775 with or without 1.5 nm gemcitabine (gem). Data are representative of at least three independent experiments. (B) The cells were treated with indicated concentrations of AZD1775 and gemcitabine for 4 days. The data are representative of at least two independent experiments. cPARP, cleaved PARP. The band intensity was quantified by imagej, and the percentage of cPARP was calculated as cPARP/(full‐length PARP + cPARP) × 100. (C) Robust cell death was induced by AZD1775–gemcitabine in the YAP‐depleted cells. The cells were treated with indicated concentration of AZD1775 and 1.5 nm gemcitabine for 4 days and analysed using flow cytometry. The data are representative of at least three independent experiments. Cells undergoing early and late apoptosis were shown in red and purple rectangles, respectively.

Yes‐associated protein activation is associated with the expression of cell cycle‐related genes 11, 12. YAP functions with E2F1 and directly upregulates its expression 13, 14. Therefore, we hypothesised that YAP inactivation leads to increased sensitivity of tumour cells to drugs targeting cell cycle or cell death regulation. To verify this, we examined the effects of YAP knockdown on the sensitivity of OVCAR‐8 cells to the several drugs. No sensitisation of OVCAR‐8 cells to dinaciclib (a pan‐CDK inhibitor), palbociclib (a CDK4/6 inhibitor) or navitoclax (a BCL family inhibitor) was observed (Fig. 1Ai–iii). However, three independent YAP siRNA sensitised OVCAR‐8 to AZD1775, a WEE1 kinase inhibitor (Fig. 1Aiv). YAP depletion itself reduced the viability of OVCAR‐8 cells, and the AZD1775 further reduced the viability efficiently, as judged by colony assay (Fig. 1B). AZD1775 abrogates cell cycle arrest upon DNA damage, and the combination of AZD1775 with cytotoxic drugs induces premature entry into mitosis with DNA damage and shows synthetic lethality 19, 22. Therefore, we studied the effects of combining AZD1775 with gemcitabine. YAP‐depleted cells were sensitive to AZD1775, and the addition of gemcitabine augmented the effect of AZD1775 (Fig. 2Ai,ii). The PARP inhibitor olaparib also augmented the effects of AZD1775, and this combination regimen induces DNA damage in leukaemia cells 23. However, this combination was less potent than AZD1775–gemcitabine therapy in OVCAR‐8 cells (Fig. S1). AZD1775‐based therapy induces robust apoptosis in various cancer models by activating apoptotic programmes 20, 23. YAP depletion increased PARP cleavage and annexin V‐positive cells induced by AZD1775–gemcitabine therapy (Fig. 2B,C). These results suggest that YAP inactivation leads to cell death by AZD1775‐based therapy in OVCAR‐8 cell line.

### AZD1775‐based therapy increases DNA damage in YAP‐depleted cells

**Figure 3:**
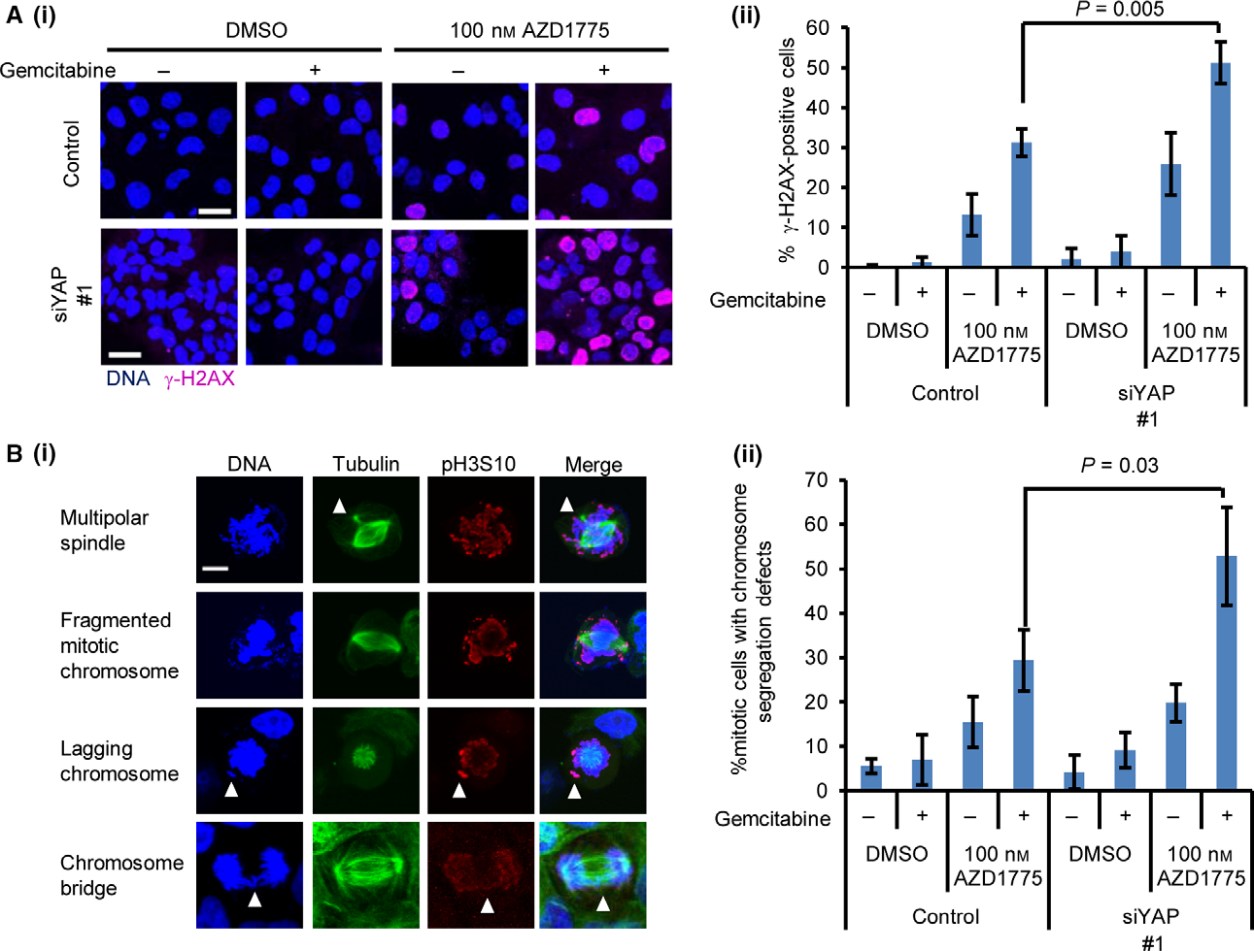
Inactivation of YAP induces γ‐H2AX accumulation and mitotic failure by AZD1775–gemcitabine therapy. (A) The YAP‐depleted cells were treated with 100 nm AZD1775 and 1.5 nm gemcitabine for 2 days. (i) Representative images. The bar represents 30 μm. (ii) The percentage of γ‐H2AX‐positive cells was calculated. The data represent the mean and standard deviation from three independent experiments. Significant differences between the groups were evaluated using an independent‐sample *t*‐test. (B) (i) Representative mitotic figures induced by AZD1775–gemcitabine. Arrowheads indicate an ectopic spindle pole, a lagging chromosome and a chromosome bridge. The bar represents 30 μm. (ii) Cells with chromosome segregation defects were counted. At least 87 cells were counted. Significant differences between the groups were evaluated using an independent‐sample *t*‐test.

In combination with cytotoxic drugs, AZD1775 increases DNA damage followed by premature entry into mitosis and cell death 27. We hypothesised that YAP depletion increases DNA damage repair and abnormal mitosis induced by AZD1775‐based therapy. We found that YAP depletion induced robust γ‐H2AX accumulation by AZD1775–gemcitabine (Fig. 3A). When combined with DNA‐damaging drugs, AZD1775 induced catastrophic mitosis by forcing cells to enter mitosis during the S phase with DNA damage 19. The cells were synchronised by a single thymidine arrest and released in the presence or absence of the AZD1775–gemcitabine combination. YAP depletion enhanced abnormal chromosome segregation, including a multipolar spindle, a fragmented mitotic chromosome, a lagging chromosome and a chromosome bridge, induced by AZD1775–gemcitabine therapy, suggesting mitosis with unrepaired chromosomes (Fig. 3B). These results suggest that YAP depletion restricts the repair of DNA damage induced by AZD1775–gemcitabine therapy and results in catastrophic mitosis. This sequence of events might explain how YAP depletion sensitises OVCAR‐8 cells to AZD1775‐based therapy.

### YAP depletion decreases gene expression of the DNA damage response components and their transcription factor E2F1

**Figure 4:**
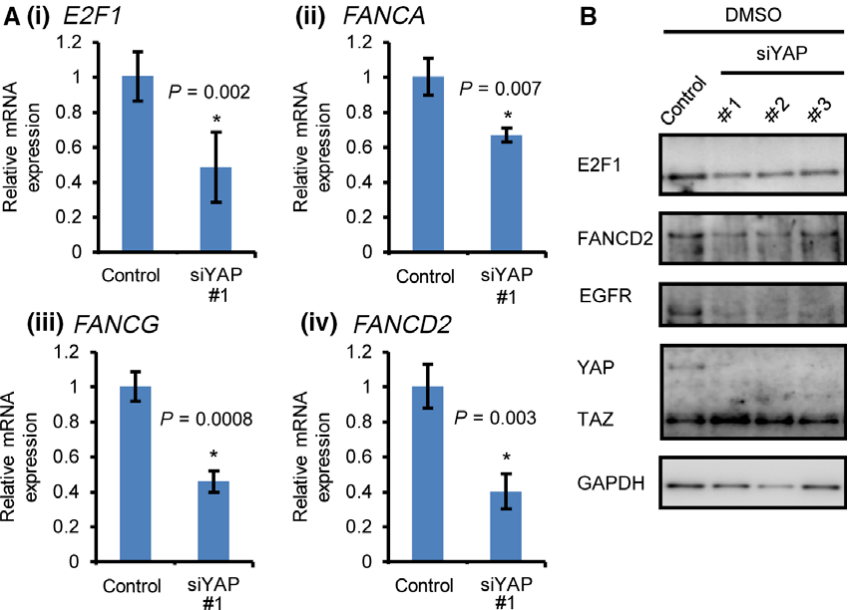
Reduced expression of the FA complex components and their transcriptional regulator E2F1 by YAP inactivation. (A) mRNA was quantified using qPCR 3 days post‐siRNA transfection. *RPSL13A* was used as a control. The data represent the mean and standard deviation of triplicates. The data are representative of at least three independent experiments. Significant differences between the groups were evaluated using an independent‐sample *t*‐test. *P < 0.05. (B) YAP was depleted using siRNA and treated with AZD1775 and gemcitabine for 4 days. Data are representative of at least two independent experiments.

Next, we investigated how YAP depletion increases DNA damage induced by AZD1775‐based therapy. YAP regulates the expression of cell cycle‐related genes including mitotic regulation, DNA replication and DNA damage responses 8, 13, 28. We hypothesised that YAP‐regulated DNA damage response genes are involved in the sensitivity to AZD1775. E2F1 transcriptionally regulates not only cell cycle‐related genes but also the DNA damage response pathway components, including the FA pathway 29. YAP binds to TEAD2 and associates with the E2F1 transcription factor‐binding site 13. In mesothelioma cell lines, YAP supports *E2F1* expression 14. Therefore, YAP is critical for E2F1‐mediated cell cycle progression. Furthermore, the siRNA screens identified FA pathway components and homologous recombination‐related genes as determinants of the sensitivity of cancer cells to AZD1775‐based therapy 25. Therefore, we focused on the effect of YAP depletion on the expression of *E2F1* and the FA pathway components as one of the pathways related to the sensitivity to AZD1775. YAP depletion reduces not only *E2F1* expression (Fig. 4Ai) but also *FANCA*,* FANCG* and *FANCD2* level (Fig. 4Aii–iv). We also found that YAP silencing reduces the protein expression of FANCD2 and E2F1 together with EGFR, a transcriptional target of YAP (Fig. 4B) 6. These results suggest that YAP is critical for the expression of the FA pathway components one of the DNA damage response pathway components related to AZD1775 sensitivity, presumably through E2F1. DNA damage response genes exemplified by FA components regulated by YAP might maintain genomic integrity and explain the increase in AZD1775‐induced DNA damage by YAP inactivation.

### YAP inactivation by dasatinib sensitises OVCAR‐8 cells to AZD1775‐based therapy

**Figure 5:**
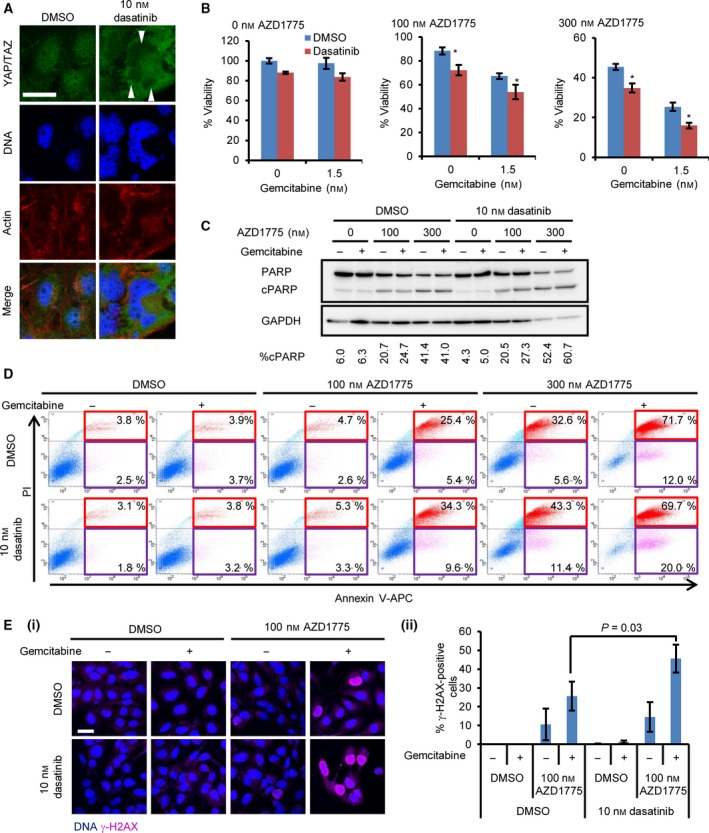
Inhibition of YAP nuclear localisation by dasatinib sensitises OVCAR‐8 cells to AZD1775‐based therapy. (A) The cells were treated with 10 nm dasatinib for 6 h and were stained with the anti‐YAP/TAZ antibody. The bar represents 30 μm. Arrowheads indicate nuclear exclusion of YAP/TAZ. (B) The cells were treated with the indicated concentration of AZD1775 with gemcitabine in the presence or absence of 10 nm dasatinib for 4 days. The data are representative of at least three independent experiments. Significant differences between the groups were evaluated using an independent‐sample *t*‐test. *P <* *0.05. (C) The cells were treated with the indicated concentration of AZD1775 and 1.5 nm gemcitabine for 4 days. The data are representative of at least two independent experiments. cPARP, cleaved PARP. The percentage of cPARP was calculated as in Fig. 2B. (D) The cells were treated with the indicated concentration of AZD1775 with or without 1.5 nm gemcitabine for 4 days. The cells were analysed using flow cytometry. The data are representative of at least two independent experiments. Cells undergoing early and late apoptosis were shown in red and purple rectangles, respectively. (E) The cells were treated with drugs for 2 days. (i) Representative images. (ii) The percentage of γ‐H2AX‐positive cells was counted. The data represent the mean and standard deviation from three independent experiments. Significant differences between the groups were evaluated using an independent‐sample *t*‐test.

We applied our findings to a possible combination therapy for ovarian cancers. We combined AZD1775‐based therapy with Food and Drug Administration (FDA)‐approved drugs. Changes in the actin cytoskeleton by the SRC inhibitor dasatinib resulted in increased phosphorylation and inhibition of the nuclear localisation of YAP 5, 26. We also found that 10 nm of dasatinib inhibited the nuclear localisation of YAP and its paralogue TAZ in OVCAR‐8 cells (Fig. 5A). We sought to use sublethal dose of dasatinib to see whether dasatinib augments the effect of AZD1775‐based therapy clearly. 10 nm of dasatinib alone was insufficient to reduce cell growth of OVCAR‐8 cells. However, this concentration of dasatinib in combination with AZD1775–gemcitabine significantly reduced the growth of OVCAR‐8 cells (Fig. 5B). The AZD1775–olaparib–dasatinib combination also reduced cell growth, although it was less potent than the AZD1775–gemcitabine–dasatinib combination (Fig. S2). AZD1775–gemcitabine–dasatinib therapy induced robust PARP cleavage and annexin V‐positive cells, whereas dasatinib alone did not induce them (Fig. 5C,D). γ‐H2AX accumulation induced by AZD1775–gemcitabine therapy was further enhanced in combination with dasatinib similar to that with YAP depletion (Fig. 5E). These results suggest that the cytoplasmic retention of YAP by dasatinib increases the efficacy of AZD1775‐based therapy, similar to YAP depletion. Therefore, combining dasatinib with AZD1775‐based therapy might be a novel therapeutic option for ovarian cancers.

## Discussion

**Figure 6:**
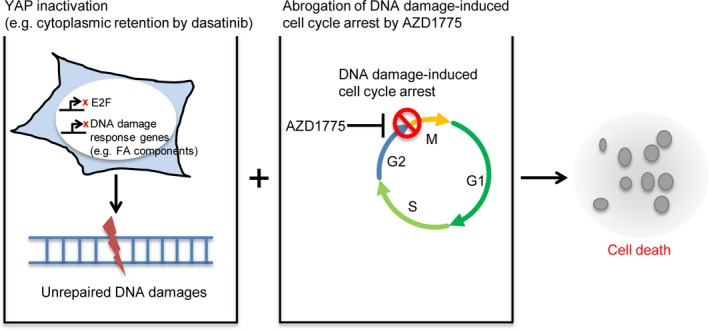
Mechanism of sensitivity to AZD1775‐based therapy through YAP inactivation. YAP inactivation (e.g. by dasatinib) reduced the expression of E2F1 and DNA damage response components exemplified by the FA pathway components. In this situation, the cells cannot tolerate the abrogation of cell cycle arrest by AZD1775 following DNA damage. This results in catastrophic mitosis and cell death.

In this study, we discovered the previously uncharacterised link between the Hippo pathway effector YAP and the sensitivity of cancer cells to a WEE1 kinase inhibitor. YAP inactivation sensitised OVCAR‐8 cells to AZD1775‐based therapy. YAP depletion increased DNA damage induced by AZD1775–gemcitabine therapy and reduced the expression of *E2F1* and the FA pathway components. Furthermore, we proposed a combination therapy using AZD1775‐based regimens and dasatinib, which inhibited the nuclear localisation of YAP. A sublethal dose of dasatinib also sensitised OVCAR‐8 cells to AZD1775‐based therapy and increased DNA damage. These results give insights into a potential new combination therapy against ovarian cancers (Fig. 6).

Yes‐associated protein depletion sensitises OVCAR‐8 cells to AZD1775‐based therapy (Fig. 2). Gemcitabine is widely used to treat ovarian cancer and augments the effect of AZD1775 22, while olaparib improves progression‐free survival for ovarian cancer patients with *BRCA1/2* mutations 30, 31 and is synergistic with AZD1775 23. We investigated AZD1775 in combination with and gemcitabine or olaparib in YAP‐depleted OVCAR‐8 cells. The combination with gemcitabine was more potent than that with olaparib (Fig. S1). Future studies are required to show the efficacy of AZD1775–olaparib therapy with YAP inactivation in multiple ovarian cancer cell lines or patient‐derived systems (xenografts, cell lines or organoids) with ‘BRCAness’. A previous study has shown that the overall response rates of AZD1775 with a DNA‐damaging agent are 43% 32, which means that this therapy cannot benefit a significant number of patients. We proposed YAP inactivation as one strategy to obtain more clinical benefits from AZD1775‐based therapy; this might result in a better response in ovarian cancer patients compared with existing monotherapies. YAP inactivation increases DNA damage induced by AZD1775‐based therapy (Fig. 3). YAP activates E2F1 transcription in order to promote cell cycle progression 13, 14. On the other hand, E2F1 directly activates *FANCA*,* FANCG* and *FANCD2* transcription 29. We identified the candidate of the determinants of the sensitivity of ovarian cancer cells to AZD1775‐based therapy. FA components are target of E2F1, and the depletion of YAP reduced the expression of FA components (Fig. 4). YAP regulates multiple target genes involved in cell cycle regulation 8, 13, 28. Therefore, other DNA damage response pathway components such as CHK1 or RAD51 might be also involved in the YAP depletion‐mediated sensitivity to AZD1775. Among them, *CHEK1* depletion is known to result in the increased sensitivity to AZD1775 25. Consistent with this, combination of CHK1 inhibitor with AZD1775 synergistically reduces cancer cell growth 33. Anyhow, the control of the expression FA components by YAP is a novel finding in this study. However, in pancreatic cancers, sensitivity to AZD1775‐based therapy is positively correlated with the proficiency, not the deficiency, of the DNA damage repair pathway 34. It is likely that the Hippo pathway target gene profiles are different in different types of tissues or cancer cells. For example, in human vascular endothelial cells, YAP regulates the transcription of cell cycle‐related genes, whereas in other cells, YAP controls the expression of *BIRC1* or other genes 35, 36. Therefore, sensitisation to AZD1775‐based therapy mediated by the YAP‐E2F1‐DNA damage response pathway might be context‐dependent. Future studies are required to identify biomarkers predicting which cancer cell types are vulnerable to AZD1775‐based therapy with YAP‐targeted therapy.

Yes‐associated protein inactivation with FDA‐approved drugs is an actionable strategy to sensitise ovarian cancer cells to AZD1775‐based therapy. To propose a novel therapeutic regimen, we utilised dasatinib, which inhibits the nuclear localisation of YAP. We found that dasatinib sensitises OVCAR‐8 cells to AZD1775‐based therapy and leads to robust accumulation of γ‐H2AX (Fig. 5). We used sublethal dose of dasatinib to observe whether there is an augmenting effect of dasatinib to AZD1775 clearly. Therefore, it is likely that the robust reduction in the viability can be observed, when higher concentration of dasatinib sufficient for fully inactivation of YAP is used. As dasatinib leads to synthetic lethality in ovarian clear cell tumours with an *ARID1A* (AT‐rich interactive domain 1A) mutation 37, our finding expands the application range of dasatinib for ovarian cancers. In non‐small‐cell lung cancer with a kinase‐inactive *BRAF* mutation, dasatinib alone induces DNA damage 38. However, we did not observe DNA damage by dasatinib alone in our experiments. This means that the DNA damage response by AZD1775, not basal‐level DNA damage, is involved in the action of dasatinib. We believe that the YAP‐induced DNA damage response is inhibited by dasatinib in OVCAR‐8 cells. Dasatinib, combined with AZD1775‐based therapy, can be a potential new therapeutic strategy involving inactivation of the YAP–E2F1–DNA damage response pathway axis. The tolerability and efficacy of AZD1775–dasatinib therapy *in vivo* are keys to applying this therapy in the future. Because YAP is involved in resistance to molecular targeted drugs 6, 7, 8, 9, our findings might expand the application of dasatinib as a chemosensitiser to subsets of molecular targeted drugs by inhibiting YAP function.

## Conclusions

Our results suggest that YAP imparts resistance to AZD1775‐based therapy through the E2F1–DNA damage response pathway axis. Dasatinib, in combination with AZD1775‐based therapy, might result in better therapeutic effects in ovarian cancers compared with AZD1775‐based therapy alone. Dasatinib might act as a chemosensitiser to molecular targeted drugs whose determinant of resistance is YAP or TAZ.

## Author contributions

YO and NN designed the study. The study was supervised by NN and YU The experiments and data analysis were performed by YO, TT, TK and NU. YS and YO wrote manuscript, and NN and YU advised it.

## Supporting information


**Fig. S1.** YAP depletion sensitised OVCAR‐8 cells to AZD1775–olaparib.
**Fig. S2.** Dasatinib sensitised OVCAR‐8 cells to AZD1775–olaparib.
