# Supplementary material for: Augmentation of the therapeutic efficacy of WEE1 kinase inhibitor AZD1775 by inhibiting the YAP–E2F1–DNA damage response pathway axis
Source: FEBS Open Bio. 2018 May 22;8(6):1001–12. doi: 10.1002/2211-5463.12440 (PMC5986022; doi:10.1002/2211-5463.12440)
Supplement: Supplementary file 1 — Fig. S1. YAP depletion sensitised OVCAR‐8 cells to AZD1775–olaparib. Fig. S2. Dasatinib sensitised OVCAR‐8 cells to AZD1775–olaparib. [file FEB4-8-1001-s001.pdf]

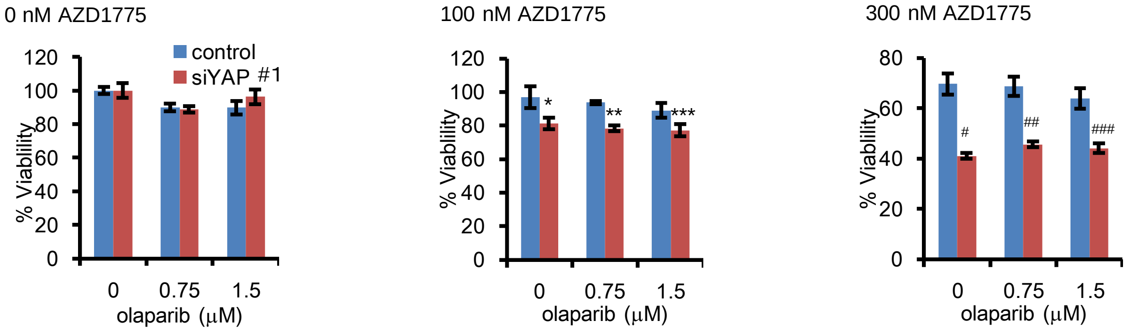

Figure S1. YAP depletion sensitized OVCAR-8 cells to AZD1775-olaparib. YAP was depleted by siRNA, and the cells were treated with the indicated amount of AZD1775 with gemcitabine for 4 days. Viability was measured by MTT assay. The data is representative of at least three independent experiments. Significant differences between the groups were evaluated using an independent *t*-test. **P*=0.02, ***P*=0.0001, ****P*=0.02, $^{\#}P$=0.0004, $^{\#\#}P$=0.0006, $^{\#\#\#}P$=0.002.

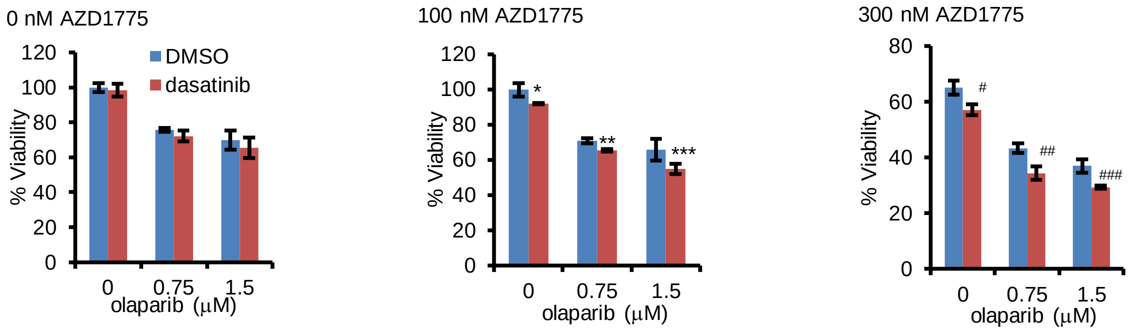

Figure S2. Dasatinib sensitized OVCAR-8 cells to AZD1775-olaparib. The cells were treated with the indicated amount of AZD1775 with gemcitabine in the presence or absence of 10 nM dasatinib for 4 days. The data is representative of at least three independent experiments. Significant differences between the groups were evaluated using an independent *t*-test. *P=0.03, **P=0.004, ***P=0.05, $^{\#}P$=0.01, $^{\#\#}P$=0.0007, $^{\#\#\#}P$=0.006.
